# The Exponentiated Lindley Geometric Distribution with Applications

**DOI:** 10.3390/e21050510

**Published:** 2019-05-20

**Authors:** Bo Peng, Zhengqiu Xu, Min Wang

**Affiliations:** 1 School of Computer Science, Southwest Petroleum University, Chengdu 610500, China; 2 Department of Mathematics and Statistics, Texas Tech University, Lubbock, TX 79409-1042, USA

**Keywords:** compounding, Lindley distribution, geometric distribution, maximum likelihood estimation, Expectation-Maximization algorithm, lifetime distribution

## Abstract

We introduce a new three-parameter lifetime distribution, the exponentiated Lindley geometric distribution, which exhibits increasing, decreasing, unimodal, and bathtub shaped hazard rates. We provide statistical properties of the new distribution, including shape of the probability density function, hazard rate function, quantile function, order statistics, moments, residual life function, mean deviations, Bonferroni and Lorenz curves, and entropies. We use maximum likelihood estimation of the unknown parameters, and an Expectation-Maximization algorithm is also developed to find the maximum likelihood estimates. The Fisher information matrix is provided to construct the asymptotic confidence intervals. Finally, two real-data examples are analyzed for illustrative purposes.

## 1. Introduction

Suppose that a company has *N* systems functioning independently and producing a certain product at a given time, where *N* is a random variable determined by economy, customers demand, etc. The reason for considering *N* as a random variable comes from a practical viewpoint, because failure (of a device for example) often occurs due to the present of an unknown number of initial defects in the system. In this paper, we consider the case in which *N* is taken to be a geometric random variable with the probability mass function given by

$$P\left(N=n\right)=\left(1-p\right)p^{n-1},$$

for 0<p<1 and *n* is a positive integer. We may take *N* to follow other discrete distributions, such as binomial, Poisson, etc, whereas they need to be truncated 0 because one must have N≥1. *Another rationale by taking N to be a geometric random variable is that the “optimum” number can be interpreted as “number to event”, matching up with the definition of a geometric random variable*, as commented by [1]. The geometric distribution has been widely used for the number of “systems” in the literature; see, for example, [2,3]. It has also been adopted to obtain some new class of distributions; see [4] for the exponential geometric (EG) distribution, [5] for the exponentiated exponential geometric (EEG) distribution, [6] for the Weibull geometric distribution, [1] for the geometric exponential Poisson (GEP) distribution, to name just a few.

On the other hand, we assume that each of *N* systems is made of α parallel components, and therefore, the system will completely shutdown if all of the components fail. Meanwhile, we assume that the failure times of the components for the *i*th system, denoted by $Z_{i1},\ldots ,Z_{i\alpha }$, are independent and identically distributed (iid) with the cumulative distribution function (cdf) G(z) and the probability density function (pdf) g(z). For simplicity of notation, let $Y_{i}$ stand for the failure time of the *i*th system and *X* denote the time to failure of the first out of the *N* functioning systems, i.e., $X=\min(Y_{1},\ldots ,Y_{N})$. Then it can be seen from [5] that the conditional cdf of *X* given *N* is given by

$$\begin{array}{cc} G(x|N) & =1-P\left(X>x|N\right)=1-{\left[1-G{\left(x\right)}^{\alpha }\right]}^{N}, \end{array}$$

and the unconditional cdf of *X* can thus be written as

(1) $$\begin{array}{cc} F(x) & =\sum_{n=1}^{\infty }G\left(x|N\right)P\left(N=n\right)=\frac{G{\left(x\right)}^{\alpha }}{1-p+p\cdot G{\left(x\right)}^{\alpha }}. \end{array}$$

The new class of distribution in (1) depends on the cdf of the failure times of the components in the system, which may follow some continuous probability distributions, such as the exponential, Lindley, and Weibull distributions. As an illustration, if the failure times of the components for the *i*th system are iid exponential random variables with the rate parameter λ, i.e., $G\left(z\right)=1-e^{-\lambda z}$, then we obtain the EEG distribution due to [5]. Its cdf is given by

(2) $$\begin{array}{c} F\left(x\right)=\frac{{\left(1-e^{-\lambda x}\right)}^{\alpha }}{1-p+p{\left(1-e^{-\lambda x}\right)}^{\alpha }}. \end{array}$$

Please note that in reliability engineering and lifetime analysis, we often assume that the failure times of the components within each system follow the exponential lifetimes; see, for example [4,5,7], among others. This assumption may be unreasonable because the hazard rate of the exponential distribution is a constant, whereas some real-life systems may not have constant hazard rates, and the components of a system are often more rigid than the system itself. Accordingly, it becomes reasonable to consider the components of a system following a distribution with a non-constant hazard function that has flexible hazard function shapes.

In this paper, we propose a new three-parameter lifetime distribution by compounding the Lindley and geometric distributions based on the new class of distribution in (1). The Lindley distribution was first proposed by [8] in the context of Bayesian statistics, as a counterexample of fiducial statistics. It has recently received considerable attention as an appropriate model to analyze lifetime data especially in applications modeling stress-strength reliability; see, for example, [9,10,11]. Ghitany et al. [12] argue that the Lindley distribution could be a better lifetime model than the exponential distribution through a numerical example and show that the hazard function of the Lindley distribution does not exhibit a constant hazard rate, indicating the flexibility of the Lindley distribution over the exponential distribution. These observations motivate us to study the structure properties of the distribution in (1) when the failure times of the units for the *i*th system are iid Lindley random variables with the parameter θ, i.e.,

(3) $$\begin{array}{c} G\left(z\right)=1-\frac{\theta +1+\theta z}{\theta +1}e^{-\theta z},\;z>0, \end{array}$$

where the parameter θ>0. Its corresponding cdf is given by

(4) $$\begin{array}{c} F\left(x\right)=\frac{{\left(1-\frac{\theta +1+\theta x}{\theta +1}e^{-\theta x}\right)}^{\alpha }}{1-p+p{\left(1-\frac{\theta +1+\theta x}{\theta +1}e^{-\theta x}\right)}^{\alpha }},\;x>0, \end{array}$$

where the parameters α>0, θ>0, and 0<p<1. We call the distribution as the *exponentiated Lindley geometric* (ELG) distribution. Indeed, it is necessary to compute the entropy measure for ELG distribution under the assumption that errors are non-Gaussian distributed (e.g., [13]). Other motivations of the ELG distribution are briefly summarized as follows. (i) It contains several lifetime distributions as special cases, such as the Lindley-geometric (LG) distribution due to [14] when α=1. (ii) It can be viewed as a mixture of exponentiated Lindley distributions introduced by [15]. (iii) The ELG distribution is a flexible model which can be widely used for modeling lifetime data in reliability and survival analysis. (iv) It exhibits monotonically increasing, decreasing, unimodal (upper-down bathtub), and bathtub shaped hazard rates but does not exhibit a constant hazard rate, which makes the ELG distribution to be superior to other lifetime distributions, which exhibit only monotonically increasing/decreasing, or constant hazard rates.

The remainder of the paper is organized as follows. In Section 2, we discuss various statistical properties of the new distribution. The maximum-likelihood estimation is considered in Section 3, and an EM algorithm is proposed to find the maximum likelihood estimates because they cannot be obtained in closed form. The maximum-likelihood estimation for censored data is also discussed briefly. In Section 4, two real-data applications are provided for illustrative purposes. Some concluding remarks are given in Section 5.

## 2. Properties of the ELG distribution

We provide statistical properties of the ELG distribution. These include the pdf and its shape (Section 2.1), hazard rate function and its shape (Section 2.2), quantile function (Section 2.3), order statistics (Section 2.4), expressions for the *n*th moments (Section 2.5), residual life function (Section 2.6), mean deviations (Section 2.7), Bonferroni and Lorenz curves (Section 2.8), and entropies (Section 2.9).

### 2.1. Probability Density function

The corresponding pdf of the ELG distribution corresponding to the cdf in (4) is given by

(5) $$\begin{array}{c} f\left(x\right)=\frac{\alpha \theta^{2}\left(1-p\right)\left(1+x\right)e^{-\theta x}{\left(1-\frac{\theta +1+\theta x}{\theta +1}e^{-\theta x}\right)}^{\alpha -1}}{\left(\theta +1\right){\left[1-p+p{\left(1-\frac{\theta +1+\theta x}{\theta +1}e^{-\theta x}\right)}^{\alpha }\right]}^{2}}, \end{array}$$

for x>0, α>0, θ>0, and 0<p<1.

It should be noted that the pdf in (5) is still a well-defined density function when p≤0. Thus, we can define the ELG distribution in (5) to any p<1. As mentioned in Section 1, the ELG distribution includes several special submodels. When α=1, it becomes the LG distribution due to [14]. When p=0 and α=1, it turns out to be the Lindley distribution due to [8]. It converges a distribution degenerating at the point 0 when $p\to 1^{-}$.

**Figure 1:**
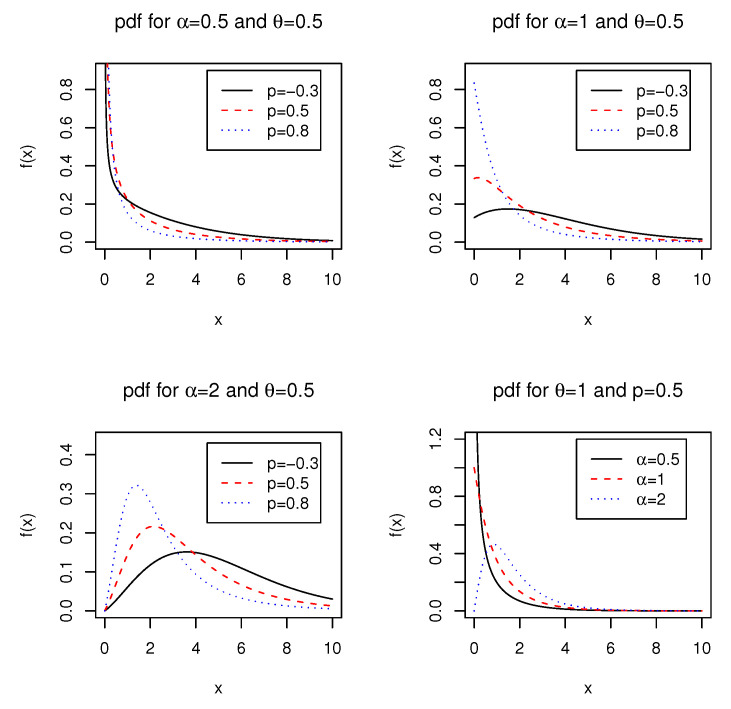
Plots of the pdf of the ELG distribution for different values of α,θ, and *p*.

Figure 1 displays the pdf of the ELG distribution in (5) with selected values of α,θ, and *p*. We observe from Figure 1 that the shape of the pdf is monotonically decreasing with the modal value of *∞* at x=0 when α<1 and the shape of the pdf appears upside-down bathtub for α>1. When α=1, we observe that the shape exhibits monotonically decreasing as well as unimodal. This observation coincides with Theorem 1 of [14], which states that *the density function of the LG distribution is (i) decreasing for all values of p and θ for which $p>\frac{1-\theta^{2}}{1+\theta^{2}}$, (ii) unimodal for all values of p and θ for which $p\leq \frac{1-\theta^{2}}{1+\theta^{2}}$*.

### 2.2. Hazard Rate Function

The failure rate function, also known as the hazard rate (hf) function, is an important characteristic for lifetime modeling. For a continuous distribution with the cdf F(x) and the pdf f(x), its failure rate function is defined as

$$h\left(x\right)=\lim_{\Delta x\to 0}=\frac{P(X<x+\Delta x|X>x)}{\Delta x}=\frac{f(x)}{S(x)},$$

where S(x)=1−F(x) is the survival function of *X*. The hf of the ELG distribution is given by

(6) $$\begin{array}{c} h\left(x\right)=\frac{\alpha \theta^{2}\left(1+x\right)e^{-\theta x}{\left(1-\frac{\theta +1+\theta x}{\theta +1}e^{-\theta x}\right)}^{\alpha -1}}{\left(\theta +1\right)\left[1-{\left(1-\frac{\theta +1+\theta x}{\theta +1}e^{-\theta x}\right)}^{\alpha }\right]\left[1-p+p{\left(1-\frac{\theta +1+\theta x}{\theta +1}e^{-\theta x}\right)}^{\alpha }\right]} \end{array}$$

for x>0, α>0, θ>0, and p<1.

**Figure 2:**
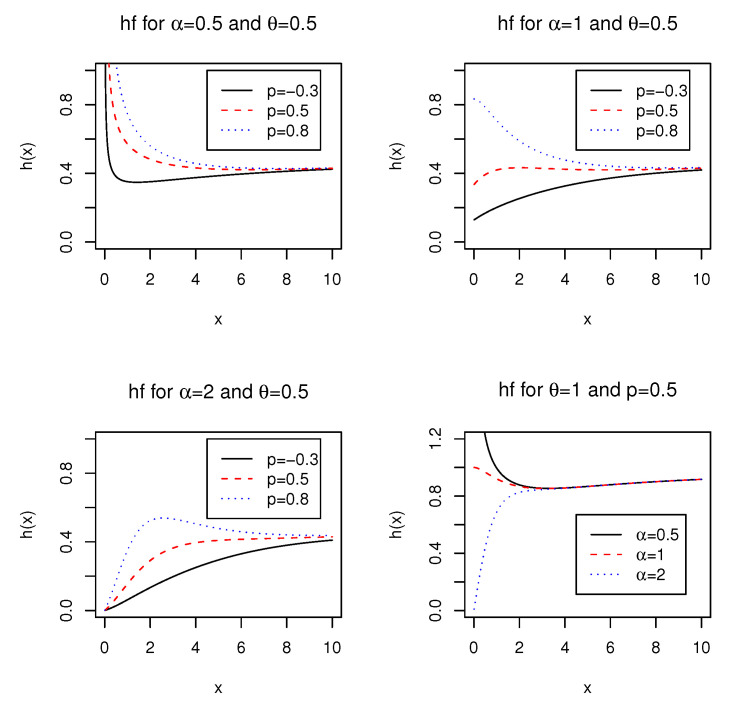
Plots of the hf of the ELG distribution for different values of α,θ, and *p*.

Figure 2 depicts shapes of the hf with selected values of α,θ, and *p*. We observe that the hf of the ELG distribution is quite flexible. For example, the shape appears monotonically decreasing if α is sufficiently small and *p* is not sufficiently large. The shape appears monotonically increasing for small *p* and large α. The shape appears bathtub-shaped or first increases then bathtub-shaped for α=1. We may conclude that the ELG distribution exhibits increasing, decreasing, upside-down bathtub, and bathtub shaped failure hazard rates, but does not exhibit a constant hazard rate.

Note also that as x→0, the initial hf behaves as $h\left(x\right)∼\left\{\alpha \theta^{2\alpha }/\left[{\left(\theta +1\right)}^{\alpha }\left(1-p\right)\right]\right\}x^{\alpha -1}$, which implies that h(0)→∞ for α<1, $h\left(0\right)=\theta^{2}/\left[\left(\theta +1\right)\left(1-p\right)\right]$ for α=1, and h(0)=0 for α>1.

### 2.3. Quantile Function

Let *Z* denote a Lindley random variable with the cdf in (3). We observe from [16] that the quantile function of the Lindley distribution is

(7) $$\begin{array}{c} G^{-1}\left(u\right)=-1-\frac{1}{\theta }-\frac{1}{\theta }W_{-1}\left(-\frac{\theta +1}{e^{\theta +1}}\left(1-u\right)\right), \end{array}$$

where 0<u<1 and $W_{-1}\left(\cdot \right)$ denotes the negative branch of the Lambert *W* function (i.e., the solution of the equation $W\left(z\right)e^{W(z)}=z$), which can be calculated by using the Lambert-W function in the R package lamW; see [17] in detail.

Let *X* be a ELG random variable with the cdf F(x) in (4). By inverting F(x)=u for 0<u<1, we obtain

$${\left(\frac{u-up}{1-up}\right)}^{1/\alpha }=1-\frac{\theta +1+\theta x}{\theta +1}e^{-\theta x}=G\left(x\right).$$

It follows from Equation (7) that the quantile function of the ELG distribution is given by

(8) $$\begin{array}{c} F^{-1}\left(u\right)=-1-\frac{1}{\theta }-\frac{1}{\theta }W_{-1}(-\frac{\theta +1}{e^{\theta +1}}[1-{\left(\frac{u-up}{1-up}\right)}^{1/\alpha }]). \end{array}$$

Please note that $-\frac{1}{e}<-\frac{\theta +1}{e^{\theta +1}}[1-{\left(\frac{u-up}{1-up}\right)}^{1/\alpha }]<0$, so the $W_{-1}\left(\cdot \right)$ is unique, which implies that $F^{-1}\left(u\right)$ is also unique. Thus, one can use Equation (8) for generating random data from the ELG distribution. In particular, the quartiles of the ELG distribution, respectively, are given by

$$\begin{array}{cc} Q_{1} & =F^{-1}\left(\frac{1}{4}\right)=-1-\frac{1}{\theta }-\frac{1}{\theta }W_{-1}(-\frac{\theta +1}{e^{\theta +1}}[1-{\left(\frac{1-p}{4-p}\right)}^{1/\alpha }]),\\ Q_{2} & =F^{-1}\left(\frac{1}{2}\right)=-1-\frac{1}{\theta }-\frac{1}{\theta }W_{-1}(-\frac{\theta +1}{e^{\theta +1}}[1-{\left(\frac{1-p}{2-p}\right)}^{1/\alpha }]),\\ Q_{3} & =F^{-1}\left(\frac{3}{4}\right)=-1-\frac{1}{\theta }-\frac{1}{\theta }W_{-1}(-\frac{\theta +1}{e^{\theta +1}}[1-{\left(\frac{3-3p}{4-3p}\right)}^{1/\alpha }]). \end{array}$$

### 2.4. Order Statistics

Suppose $X_{1},\ldots ,X_{n}$ is a random sample from the ELG distribution. Let $X_{\left(1\right)}<X_{\left(2\right)}<\cdots <X_{\left(n\right)}$ be the corresponding order statistics. The pdf for the *r*th order statistic of the ELG distribution, say $Y=X_{\left(r\right)}$, is given by

fY(y)=n!(r−1)!(n−r)!Fr−1(y)1−F(y)n−rf(y)=n!(r−1)!(n−r)!∑τ=0n−rn−rτ(−1)τFr−1+τ(y)f(y)=αθ2(1−p)(1+x)e−θxn!(θ+1)2(r−1)!(n−r)!∑τ=0n−rn−rτ(−1)τ1−θ+1+θxθ+1e−θxα(r+τ)−11−p+p1−θ+1+θxθ+1e−θxαr+τ+1.

The corresponding cdf of *Y* is given by

FY(y)=∑j=rnFj(y)1−F(y)n−j=∑j=rn∑τ=0n−jnjn−jτ(−1)τFj+τ(y)=∑j=rn∑τ=0n−jnjn−jτ(−1)τ1−θ+1+θxθ+1e−θxα(j+τ)1−p+p1−θ+1+θxθ+1e−θxαj+τ.

In practice, we may be interested in studying the asymptotic distribution of the extreme values $X_{\left(1\right)}$ and $X_{\left(n\right)}$. By using L’Hospital’s rule, we have

$$\begin{array}{cc} \lim_{t\to \infty }\frac{1-F(t+x/\theta )}{1-F(t)} & =\lim_{t\to \infty }\frac{f(t+x/\theta )}{f(t)}=\frac{1-{\left[1-\frac{\theta t}{\theta +1}e^{-(\theta t+x)}\right]}^{\alpha }}{1-{\left[1-\frac{\theta t}{\theta +1}e^{-\theta t}\right]}^{\alpha }}\\ & =e^{-x}. \end{array}$$

In addition, by using L’Hospital’s rule, it can be easily shown that

$$\begin{array}{cc} \lim_{t\to 0}\frac{F(tx)}{F(t)} & =\lim_{t\to 0}\frac{xf(xt)}{f(t)}=\lim_{t\to 0}{\left(\frac{1-\frac{\theta +1+\theta tx}{\theta +1}e^{-\theta tx}}{1-\frac{\theta +1+\theta t}{\theta +1}e^{-\theta t}}\right)}^{\alpha }\\ & =x^{\alpha }. \end{array}$$

By following Theorem 1.6.2 in [18] we observe that there must be some normalizing constants $a_{n}>0$, $b_{n}$, $c_{n}>0$, and $d_{n}$, such that

$$Pr\left[a_{n}\left(X_{\left(1\right)}-b_{n}\right)\leq x\right]\to \exp\left(-e^{-x}\right)$$

and

$$Pr\left[c_{n}\left(X_{\left(n\right)}-d_{n}\right)\leq x\right]\to 1-\exp\left(-x^{a}\right)$$

as n→∞. The form of the normalizing constants can be determined by using Corollary 1.6.3 in [18]. As an illustration, one can see that $a_{n}=\theta$ and $b_{n}=F^{-1}\left(1-1/n\right)$, where $F^{-1}\left(\cdot \right)$ denotes the inverse function of F(·).

### 2.5. Moment Properties

Many important features of a distribution can be characterized through its moments, such as dispersion, skewness, and kurtosis. To derive the *n*th moment of the ELG distribution, we consider the Taylor series expansion of the form

(9) (1+x)−a=∑k=0∞−akxk,

which converges for |x|<1. This provides that

(10) 1−p+pGα(x)−1=∑k=0∞−1k−p1−Gα(x)k=∑k=0∞∑j=0k−1kkj(−1)j+kpkG(x)αj,

where −1k is the generalized binomial coefficient. Therefore, we can rewrite Equation (4) as

(11) F(x)=∑k=0∞∑j=0k−1kkj(−1)j+kpkG(x)αj+α=∑k=0∞∑j=0k−1kkj(−1)j+kpk1−θ+1+θxθ+1e−θxαj+α.

We observe that the ELG distribution is a mixture of exponentiated Lindley distributions introduced by [15], i.e.,

1−p+pGα(x)−1=11−p1+p1−pGα(x)−1=11−p∑k=0∞−1kp1−pGα(x)k,

which is convergent for $|p/\left(1-p\right)G^{\alpha }\left(x\right)|<1$. They show that if *Y* is an exponentiated Lindley random variable with parameters θ and β, the *n*th moment and the moment generating function of *Y* are, respectively, given by

$$I\;E\left(Y_{\theta ,\beta }^{n}\right)=\frac{\beta \theta^{2}}{1+\theta }K\left(\beta ,\theta ,n,\theta \right)$$

and

$$M_{Y_{\theta ,\beta }}\left(t\right)=\frac{\beta \theta^{2}}{1+\theta }K\left(\beta ,\theta ,0,\theta -t\right)$$

for t<θ, where

K(a,b,c,δ)=∫0∞xc(1+x)1−1+b+bx1+be−bxa−1e−δxdx=∑i=0∞∑j=0i∑k=0j+1a−1iijj+1k(−1)ibjΓ(c+k+1)(1+b)i(bi+δ)c+k+1.

By using Equation (11), we obtain the *n*th moment of *X* can be rewritten as

(12) μr(x)=IE(Xn)=∑k=0∞−1k(−p)k∑j=0kkj(−1)jIEYθ,αj+αn=∑k=0∞−1k(−p)k∑j=0kkj(−1)j(αj+α)θ21+θK(αj+α,θ,n,θ)=αθ21+θ∑k=0∞∑j=0k−1kkj(−1)j+kpk(j+1)K(αj+α,θ,n,θ)

for n=1,2,…. Equation (12) can be adopted to compute the third and fourth central moments of the ELG distribution, which are then used to define skewness and kurtosis, respectively. For instance, based on the first four moments of the ELG distribution, the measures of skewness γ and kurtosis κ of the ELG distribution are, respectively, given by

$$\gamma =\frac{\mu_{3}\left(x\right)-3\mu_{2}\left(x\right)\mu_{2}\left(x\right)+2\mu_{1}^{3}\left(x\right)}{{\left[\mu_{2}\left(x\right)-\mu_{1}^{2}\left(x\right)\right]}^{3/2}},$$

and

$$\kappa =\frac{\mu_{4}\left(x\right)-4\mu_{1}\left(x\right)\mu_{3}\left(x\right)+6\mu_{1}^{2}\left(x\right)\mu_{2}\left(x\right)-3\mu_{1}^{4}\left(x\right)}{{\left[\mu_{2}\left(x\right)-\mu_{1}^{2}\left(x\right)\right]}^{2}}.$$

The moment generating function of the ELG distribution, denoted by $M_{X}\left(t\right)$, is given by

MX(t)=∑k=0∞−1k(−p)k∑j=0kkj(−1)jMYθ,αj+α(t)=αθ21+θ∑k=0∞∑j=0k−1kkj(−1)j+kpk(j+1)K(αj+α,θ,0,θ−t).

Thereafter, we can use $M_{X}\left(t\right)$ to obtain the *n*th moment about zero of the ELG distribution. In particular, if $|\frac{p}{1-p}G^{\alpha }\left(x\right)|<1$, then Equation (11) can be simplified to

(13) $$F\left(x\right)=\frac{1}{1-p}\sum_{k=0}^{\infty }\frac{{\left(-p\right)}^{k}}{{\left(1-p\right)}^{k}}{\left(1-\frac{\theta +1+\theta x}{\theta +1}e^{-\theta x}\right)}^{\alpha k+\alpha }.$$

The corresponding *n*th moment of *X* can be simplified as

(14) $$\begin{array}{cc} \mu_{r}\left(x\right)=I\;E\left(X^{n}\right) & =\frac{1}{1-p}\sum_{k=0}^{\infty }\frac{{\left(-p\right)}^{k}}{{\left(1-p\right)}^{k}}I\;E\left(Y_{\theta ,\alpha k+\alpha }^{n}\right)\\ & =\frac{\alpha \theta^{2}}{\left(1+\theta )(1-p\right)}\sum_{k=0}^{\infty }\frac{{\left(-p\right)}^{k}\left(j+1\right)}{{\left(1-p\right)}^{k}}K\left(\alpha k+\alpha ,\theta ,n,\theta \right) \end{array}$$

for n=1,2,⋯, and the moment generating function of the ELG distribution is given by

$$\begin{array}{cc} M_{X}\left(t\right) & =\frac{1}{1-p}\sum_{k=0}^{\infty }\frac{{\left(-p\right)}^{k}}{{\left(1-p\right)}^{k}}M_{Y_{\theta ,\alpha k+\alpha }}\left(t\right)\\ & =\frac{\alpha \theta^{2}}{\left(1+\theta )(1-p\right)}\sum_{k=0}^{\infty }\frac{{\left(-p\right)}^{k}\left(j+1\right)}{{\left(1-p\right)}^{k}}K\left(\alpha k+\alpha ,\theta ,0,\theta -t\right). \end{array}$$

### 2.6. Residual Life Function

Given that a component of a system survives up to time t≥0, the residual life will be the period beyond *t* until the time of failure occurs in the system and is thus defined by the conditional random variable X−t∣X>t. The mean residual life plays an important role in survival analysis and reliability of characterizing lifetime, because it can be used to determine a unique corresponding lifetime distribution. The *r*th moment of the residual life of the ELG distribution can be obtained by the general formula

(15) $$m_{r}\left(t\right)=I\;E\left[{\left(X-t\right)}^{r}|Y>t\right]=\frac{1}{S(t)}\int_{t}^{\infty }{\left(x-t\right)}^{r}f\left(x\right)\;dx,$$

where S(t)=1−F(t) is the survival function defined before. Noting that the ELG distribution is a mixture of exponentiated Lindley distributions, we may calculate $m_{r}\left(t\right)$ by using the expression in Lemma 2 of [15], which is given by

L(a,b,c,t)=∫t∞xc(1+x)1−b+1+bxb+1e−bxa−1e−bxdx=∑i=1∞∑j=1i∑k=0j+1a−1iijj+1k(−1)ibjΓ(c+k+1,(bi+b)t)(1+b)i(bi+b)c+k+1,

where $\Gamma \left(a,x\right)=\int_{x}^{\infty }t^{a-1}\exp\left(-t\right)\;dx$ represents the complementary incomplete gamma function. Let *X* be an ELG random variable. By using the Taylor series expansion in (9), it can be easily shown that

∫t∞xrf(x)dx=αθ2(1−p)θ+1∫t∞xr(1+x)1−θ+1+θxθ+1e−θxα−11−p+p1−θ+1+θxθ+1e−θxα−2dx=αθ2(1−p)θ+1∫t∞∑l=0∞−2l(−p)l∑j=0llj(−1)jxr(1+x)1−θ+1+θxθ+1e−θxα+αj−1dx=αθ2(1−p)θ+1∑l=0∞−2l(−p)l∑j=0llj(−1)j∫t∞xr(1+x)1−θ+1+θxθ+1e−θxα+αj−1dx=αθ2(1−p)θ+1∑l=0∞∑j=0l−2llj(−1)j+lplL(α+αj,θ,r,t).

From the binomial expansion for ${\left(x-t\right)}^{r}$, we get that the *r*th order moment of the residual life of the ELG distribution is given by

mr(t)=1S(t)∫t∞(x−t)rf(x)=1S(t)∫t∞∑k=0rrkxr−k(−t)kf(x)dx=1S(t)∑k=0rrk(−t)k∫t∞xr−kf(x)dx=1S(t)αθ2(1−p)θ+1∑l=0∞∑j=0l∑k=0rrk−2llj(−1)j+l+ktkplL(α+αj,θ,r−k,t).

The mean and variance of the residual life function of the ELG distribution can be easily obtained using $m_{1}\left(t\right)$ and $m_{2}\left(t\right)$, and are not shown here for simplicity. In a similar way as done for Equation (13), it can be shown that if $|\frac{p}{1-p}G^{\alpha }\left(x\right)|<1$, then

(16) ∫t∞xrf(x)dx=αθ2(θ+1)(1−p)∑l=0∞−2l(−p)l(1−p)lL(α+αl,θ,r,t),

and the *r*th order moment of the residual life of the ELG distribution can be written as

mr(t)=1S(t)αθ2(θ+1)(1−p)∑l=0∞∑k=0rrk−2l(−1)k+lpltk(1−p)lL(α+αl,θ,r−k,t).

### 2.7. Mean Deviations

We consider the totality of deviations from the mean and median and the mean deviation from the mean, which is often used to estimate the amount of scatter in a population. The mean deviation is a more robust statistic to outliers in the data set than the standard deviation and the mean deviation from the median is a measure of statistical dispersion, which is a more robust statistic to outliers than the sample variance or standard deviation.

Let *X* denote a random variable with the pdf f(x), the cdf F(x), mean μ, and median *M*. The mean deviation about the mean and the mean deviation about the median are defined by

δ1(X)=∫0∞|x−μ|f(x)dx=∫0μ(μ−x)f(x)dx+∫μ∞(x−μ)f(x)dx=μF(μ)−∫0μxf(x)dx+∫μ∞xf(x)dx−μ(1−F(μ))=2μF(μ)−2μ+2∫μ∞xf(x)dx=2μF(μ)−2μ+2αθ2(1−p)θ+1∑l=0∞∑j=0l−2llj(−1)j+lplL(α+αj,θ,1,μ)

and

δ2(X)=∫0∞|x−M|f(x)dx=∫0M(M−x)f(x)dx+∫M∞(x−M)f(x)dx=MF(M)−∫0Mxf(x)dx+∫M∞xf(x)dx−M(1−F(M))=μ+2∫M∞xf(x)dx=−μ+2αθ2(1−p)θ+1∑l=0∞∑j=0l−2llj(−1)j+lplL(α+αj,θ,1,M).

respectively. Of particular note is that when $|\frac{p}{1-p}G^{\alpha }\left(x\right)|<1$, the mean deviations above can be further simplified as

δ1(X)=2μF(μ)−2μ+2αθ2(1−p)(θ+1)∑i=1∞−2jpp+1jL(α+αj,θ,1,t).

and

δ2(X)=−μ+2αθ2(1−p)(θ+1)∑i=1∞−2jpp+1jL(α+αj,θ,1,M).

### 2.8. Bonferroni and Lorenz Curves

The Bonferroni and Lorenz curves (Bonferroni 1930) have many practical applications not only in economics and poverty, but also in other fields like reliability, lifetime testing, insurance, and medicine. For a random variable *X* with cdf F(·), the Bonferroni and Lorenz curves are defined by

(17) $$B\left[F(x)\right]=\frac{1}{\mu F(x)}\int_{0}^{q}xf\left(x\right)\;dx,$$

where μ=IE(X), and

(18) $$L\left[F(x)\right]=\frac{1}{F(x)}\int_{0}^{q}xf\left(x\right)\;dx,$$

respectively. If *X* is an ELG random variable with the pdf in (5), we observe Equation (17) can be written as

BF(x)=1μF(x)∫0qxf(x)dx=1μF(x)∫0∞xf(x)dx−∫q∞xf(x)dx=1μF(x)μ−αθ2(1−p)θ+1∑l=0∞∑j=0l−2llj(−1)j+lplL(α+αj,θ,1,q),

which is obtained by using Equation (16) with t=q and r=1. By using Equation (18), it follows easily that the Lorenz curve of the ELG distribution is given by L[F(x)]=μB[F(x)].

### 2.9. Entropies

It is well known that an entropy of a random variable *X* is a measure of variation of the uncertainty. The Rényi entropy is defined as

$$\begin{array}{c} I_{R}\left(\gamma \right)=\frac{1}{\gamma }\log\int_{0}^{\infty }f^{\gamma }\left(x\right)\;dx, \end{array}$$

where γ>0 and γ≠1. The Shannon entropy is defined as E$\left[-\log(f(x))\right]$, which is a particular case of the Rényi entropy as γ→1. We first observe that

∫0∞fγ(x)dx=αθ2(1−p)1+θ0γ∞(1+x)γe−θγx1−θ+1+θxθ+1e−θxαγ−γ1−p+p1−θ+1+θxθ+1e−θxα−2γdx=αθ2(1−p)1+θγ∑k=0∞∑j=0∞−2γkkj(−1)k+jp0k∞(1+x)γe−θγx1−θ+1+θxθ+1e−θxαγ−γ+αjdx,

which shows that the Rényi entropy of the ELG distribution is given by

IR(γ)=1γlog∫0∞fγ(x)dx,=γ1−γlogαθ2(1−p)1+θ+11−γlog∑k=0∞∑j=0∞−2γkkj(−1)k+jpk∫0∞(1+x)γe−θγx1−θ+1+θxθ+1e−θxαγ−γ+αjdx.

**Figure 3:**
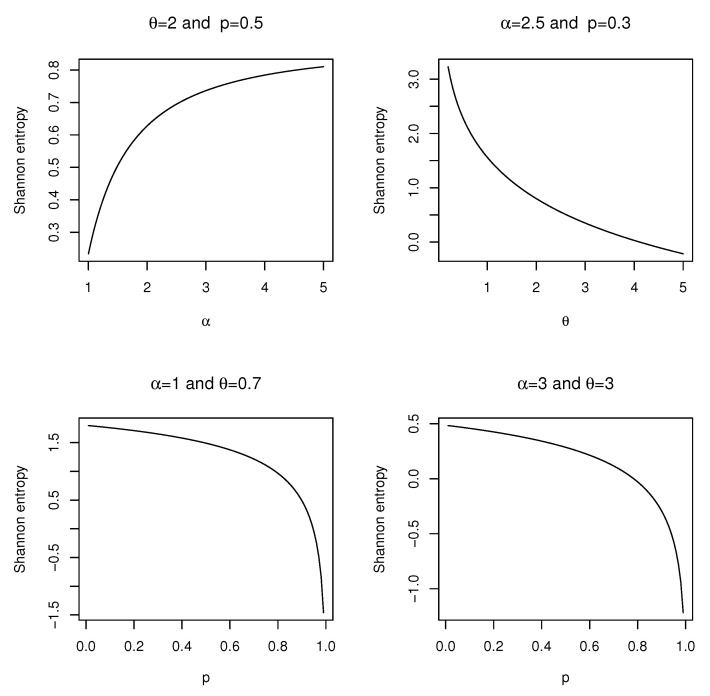
Plots of the Shannon entropy of the ELG distribution for different values of α,θ, and *p*.

It can be shown that the Shannon entropy of the ELG distribution is given by

$$\begin{array}{cc} H(X) & =E[-\log f(X)]\\ & =-\log\left[\frac{\alpha \theta^{2}\left(1-p\right)}{1+\theta }\right]-E\left[\log\left(1+x\right)\right]+\theta E\left[x\right]-\left(\alpha -1\right)E\left[\log\left(G\left(x\right)\right)\right]+2E[log\left(1-p+pG^{\alpha }\left(x\right)\right], \end{array}$$

which can be easily evaluated using a unidimensional integral. Figure 3 depicts shapes of the Shannon entropy of the ELG distribution with several selected values of α,θ, and *p*. It deserves mentioning that the entropy measure of the ELG distribution can be estimated by using numerical integration methods with the (plug-in) estimators found in the following section.

## 3. Estimation of Parameters

We adopt the maximum likelihood estimation to estimate the unknown parameters (Section 3.1) and develop an Expectation-Maximization (EM) algorithm to find the maximum likelihood estimate (MLE) (Section 3.2). We also discuss the MLEs of the unknown parameters when the data is censored (Section 3.3).

### 3.1. Maximum Likelihood Estimation

It is well known that the MLE is often used to estimate the unknown parameter of a distribution because of its attractive properties, such as consistency, asymptotic normality, etc. Let $X_{1},\ldots ,X_{n}$ be a random sample from the ELG distribution with unknown parameter vector ϕ=(θ,α,p). Then the log-likelihood function l=l(ϕ;x) is given by

(19) $$\begin{array}{cc} l= & \;n\log\alpha +2n\log\theta -n\log\left(\theta +1\right)+n\log\left(1-p\right)+\sum_{i=1}^{n}\log(1+x_{i})-\theta \sum_{i=1}^{n}x_{i}+\left(\alpha -1\right)\\ & \;\times \sum_{i=1}^{n}\log\left(1-\frac{\theta +1+\theta x_{i}}{\theta +1}e^{-\theta x_{i}}\right)-2\sum_{i=1}^{n}\log\left[1-p+p{\left(1-\frac{\theta +1+\theta x_{i}}{\theta +1}e^{-\theta x_{i}}\right)}^{\alpha }\right]. \end{array}$$

For notational convenience, let

$$\tau_{i}\left(\theta \right)=1-\frac{\theta +1+\theta x_{i}}{\theta +1}e^{-\theta x_{i}},$$

for i=1,…,n. The MLEs of the unknown parameters can be obtained by taking the first partial derivatives of Equation (19) with respect to α, θ, and *p* and putting them equal to 0. We have the following likelihood equations

(20) $$\begin{array}{cc} \frac{\partial l}{\partial \alpha } & =\frac{n}{\alpha }+\sum_{i=1}^{n}\log\left[\tau_{i}\left(\theta \right)\right]-2p\sum_{i=1}^{n}\frac{\tau_{i}^{\alpha }\left(\theta \right)\log\left[\tau_{i}\left(\theta \right)\right]}{1-p+p\tau_{i}^{\alpha }\left(\theta \right)}, \end{array}$$

(21) $$\begin{array}{cc} \frac{\partial l}{\partial \theta } & =\frac{2n}{\theta }-\frac{n}{\theta +1}-\sum_{i=1}^{n}x_{i}+\frac{(\alpha -1)\theta }{{\left(\theta +1\right)}^{2}}\sum_{i=1}^{n}\frac{x_{i}\left(2+\theta +\theta x_{i}+x_{i}\right)e^{-\theta x_{i}}}{\tau_{i}\left(\theta \right)}-\frac{2\alpha p\theta }{{\left(\theta +1\right)}^{2}}\\ & \;\times \sum_{i=1}^{n}\frac{\tau_{i}^{\alpha -1}\left(\theta \right)x_{i}\left(2+\theta +\theta x_{i}+x_{i}\right)e^{-\theta x_{i}}}{1-p+p\tau_{i}^{\alpha }\left(\theta \right)}, \end{array}$$

(22) $$\begin{array}{cc} \frac{\partial l}{\partial p} & =-\frac{n}{1-p}+2\sum_{i=1}^{n}\frac{1-\tau_{i}^{\alpha }\left(\theta \right)}{1-p+p\tau_{i}^{\alpha }\left(\theta \right)}. \end{array}$$

Please note that the MLEs, respectively $\hat{\alpha }$, $\hat{\theta }$ and $\hat{p}$ of α, θ and *p* cannot be solved analytically. Numerical iteration techniques, such as the Newton-Raphson algorithm, are required to solve these equations, whereas the second derivatives of the log-likelihood are required for all iterations involved in numerical iteration techniques. We thus develop an EM algorithm to estimate the MLEs of the unknown parameters.

For interval estimation of the parameters, we consider suitable pivotal quantities based on the asymptotic properties of the MLEs and approximate the distributions of these quantities by the normal distribution. We observe that

$$\begin{array}{cc} \frac{\partial^{2}\log l}{\partial \alpha^{2}} & =-\frac{n}{\alpha^{2}}-2p\left(1-p\right)\sum_{i=1}^{n}\frac{\tau_{i}^{\alpha }\left(\theta \right){\left[\log\left(\tau_{i}\left(\theta \right)\right)\right]}^{2}}{{\left[1-p+p\tau_{i}^{\alpha }\left(\theta \right)\right]}^{2}},\\ \frac{\partial^{2}\log l}{\partial \theta^{2}} & =-\frac{2n}{\theta^{2}}+\frac{n}{{\left(\theta +1\right)}^{2}}-\frac{\left(\alpha -1\right)}{{\left(\theta +1\right)}^{4}}\sum_{i=1}^{n}\frac{x_{i}e^{-\theta x_{i}}\left[e^{-\theta x_{i}}\left(x_{i}+2x_{i}\theta +2+2\theta \right)+\left(t+1\right)\kappa_{i}\right]}{\tau_{i}^{2}\left(\theta \right)}\\ & +\frac{2\alpha p\theta^{2}}{{\left(\theta +1\right)}^{4}}\sum_{i=1}^{n}\frac{x_{i}^{2}{\left(2+\theta +\theta x_{i}+x_{i}\right)}^{2}e^{-2\theta x_{i}}\tau_{i}^{\alpha -2}\left(\theta \right)\left\{\left(1-\alpha \right)\left[1-p+p\tau_{i}^{\alpha }\left(\theta \right)\right]+\alpha p\tau_{i}^{\alpha }\left(\theta \right)\right\}}{{\left[1-p+p\tau_{i}^{\alpha }\left(\theta \right)\right]}^{2}}\\ & +\frac{2\alpha p}{{\left(\theta +1\right)}^{3}}\sum_{i=1}^{n}\frac{x_{i}\kappa_{i}\tau_{i}^{\alpha -1}\left(\theta \right)e^{-\theta x_{i}}}{1-p+p\tau_{i}^{\alpha }\left(\theta \right)}, \end{array}$$

$$\begin{array}{cc} \frac{\partial^{2}\log l}{\partial p^{2}} & =-\frac{n}{{\left(1-p\right)}^{2}}+2\sum_{i=1}^{n}{\left[\frac{1-\tau_{i}^{\alpha }\left(\theta \right)}{1-p+p\tau_{i}^{\alpha }\left(\theta \right)}\right]}^{2},\\ \frac{\partial^{2}\log l}{\partial \alpha \partial \theta } & =\frac{\partial^{2}\log l}{\partial \theta \partial \alpha }=\frac{\theta }{{\left(\theta +1\right)}^{2}}\sum_{i=1}^{n}\frac{x_{i}\left(2+\theta +\theta x_{i}+x_{i}\right)e^{-\theta x_{i}}}{\tau_{i}\left(\theta \right)}-\frac{2p\theta }{{\left(\theta +1\right)}^{2}}\\ & \;\times \sum_{i=1}^{n}\frac{\left[\alpha \left(1-p\right)\log\left(\tau_{i}\left(\theta \right)\right)+1-p+p\tau_{i}^{\alpha }\left(\theta \right)\right]x_{i}\left(2+\theta +\theta x_{i}+x_{i}\right)e^{-\theta x_{i}}\tau_{i}^{\alpha -1}\left(\theta \right)}{{\left[1-p+p\tau_{i}^{\alpha }\left(\theta \right)\right]}^{2}},\\ \frac{\partial^{2}\log l}{\partial \alpha \partial p} & =\frac{\partial^{2}\log l}{\partial p\partial \alpha }=-\frac{\tau_{i}^{\alpha }\left(\theta \right)\log\left(\tau_{i}\left(\theta \right)\right)}{{\left[1-p+p\tau_{i}\left(\theta \right)\right]}^{2}},\\ \frac{\partial^{2}\log l}{\partial \theta \partial p} & =\frac{\partial^{2}\log l}{\partial p\partial \theta }=-2\frac{\tau_{i}^{\alpha }\left(\theta \right)\log\left(\tau_{i}\left(\theta \right)\right)}{{\left[1-p+p\tau_{i}^{\alpha }\left(\theta \right)\right]}^{2}}, \end{array}$$

where $\kappa_{i}=\left(\theta^{3}+\theta \right)\left(x_{i}+x_{i}^{2}\right)+\theta^{2}\left(3x_{i}+2x_{i}^{2}\right)-x_{i}-2$ for i=1,…,n. The observed Fisher information matrix of α, θ, and *p* can be written as

$$\begin{array}{c} I=-\left(\begin{array}{ccc} \frac{\partial^{2}\log l}{\partial \alpha^{2}} & \frac{\partial^{2}\log l}{\partial \alpha \partial \theta } & \frac{\partial^{2}\log l}{\partial \alpha \partial p}\\ \frac{\partial^{2}\log l}{\partial \theta \partial \alpha } & \frac{\partial^{2}\log l}{\partial \theta^{2}} & \frac{\partial^{2}\log l}{\partial \theta \partial p}\\ \frac{\partial^{2}\log l}{\partial p\partial \alpha } & \frac{\partial^{2}\log l}{\partial p\partial \theta } & \frac{\partial^{2}\log l}{\partial p^{2}} \end{array}\right), \end{array}$$

so the variance-covariance matrix of the MLEs $\hat{\alpha }$, $\hat{\theta }$ and $\hat{p}$ may be approximated by inverting the matrix I and is thus given by

$$V=-{\left(\begin{array}{ccc} \frac{\partial^{2}\log l}{\partial \alpha^{2}} & \frac{\partial^{2}\log l}{\partial \alpha \partial \theta } & \frac{\partial^{2}\log l}{\partial \alpha \partial p}\\ \frac{\partial^{2}\log l}{\partial \theta \partial \alpha } & \frac{\partial^{2}\log l}{\partial \theta^{2}} & \frac{\partial^{2}\log l}{\partial \theta \partial p}\\ \frac{\partial^{2}\log l}{\partial p\partial \alpha } & \frac{\partial^{2}\log l}{\partial p\partial \theta } & \frac{\partial^{2}\log l}{\partial p^{2}} \end{array}\right)}^{-1}=\left(\begin{array}{ccc} var(\alpha ) & cov(\alpha ,\theta ) & cov(\alpha ,p)\\ cov(\theta ,\alpha ) & var(\theta ) & cov(\theta ,p)\\ cov(p,\alpha ) & cov(p,\theta ) & var(p) \end{array}\right).$$

The asymptotic joint distribution of the MLEs $\hat{\alpha }$, $\hat{\theta }$, and $\hat{p}$ can be treated as being approximately multivariate normal and is given by

(23) $$\begin{array}{c} \left(\begin{array}{c} \hat{\alpha }\\ \hat{\theta }\\ \hat{p} \end{array}\right)∼N\left[\left(\begin{array}{c} \alpha \\ \theta \\ p \end{array}\right),\;\;\left(\begin{array}{ccc} var(\alpha ) & cov(\alpha ,\theta ) & cov(\alpha ,p)\\ cov(\theta ,\alpha ) & var(\theta ) & cov(\theta ,p)\\ cov(p,\alpha ) & cov(p,\theta ) & var(p) \end{array}\right)\right]. \end{array}$$

Since V involves the unknown parameters α, θ, and *p*, we replace these parameters by their corresponding MLEs to obtain an estimate of V denoted by

$$\hat{V}=\left(\begin{array}{ccc} \hat{var(\alpha )} & \hat{cov(\alpha ,\theta )} & \hat{cov(\alpha ,p)}\\ \hat{cov(\theta ,\alpha )} & \hat{var(\theta )} & \hat{cov(\theta ,p)}\\ \hat{cov(p,\alpha )} & \hat{cov(p,\theta )} & \hat{var(p)} \end{array}\right).$$

The asymptotic 100(1−γ)% confidence intervals of α, θ, and *p* are determined by

$$\begin{array}{c} \left[\hat{\alpha }-z_{\gamma /2}\sqrt{\hat{var(\alpha )}},\;\hat{\alpha }+z_{\gamma /2}\sqrt{\hat{var(\alpha )}}\right],\\ \left[\hat{\theta }-z_{\gamma /2}\sqrt{\hat{var(\theta )}},\;\hat{\theta }+z_{\gamma /2}\sqrt{\hat{var(\theta )}}\right],\\ \left[\hat{p}-z_{\gamma /2}\sqrt{\hat{var(p)}},\;\hat{p}+z_{\gamma /2}\sqrt{\hat{var(p)}}\right], \end{array}$$

respectively, where $z_{p}$ is the upper *p*th percentile of the standard normal distribution.

The likelihood ratio (LR) can be used to evaluate the difference between the ELG distribution and its special submodels. We partition the parameters of the ELG distribution into ${\left(\phi_{1}^{\prime },\phi_{2}^{\prime }\right)}^{\prime }$, where $\phi_{1}$ is the parameter of interest and $\phi_{2}$ is the remaining parameters. Consider the hypotheses

(24) $$H_{0}:\phi_{1}=\phi_{1}^{\left(0\right)}\;\mathrm{versus}\;H_{1}:\phi_{1}\neq \phi_{1}^{\left(0\right)}.$$

The LR statistic for the test of the null hypothesis in (24) is given by

(25) $$\omega =2\left\{l\left(\hat{\phi };x\right)-l\left({\hat{\phi }}^{*};x\right)\right\},$$

where $\hat{\phi }$ and ${\hat{\phi }}^{*}$ are the restricted and unrestricted maximum likelihood estimators under $H_{0}$ and $H_{1}$, respectively. Under $H_{0}$, it follows

(26) $$\begin{array}{c} \omega \overset{D}{⟶}\chi_{\kappa }^{2}, \end{array}$$

where $\overset{D}{⟶}$ denotes convergence in distribution as n→∞ and κ is the dimension of the subset $\phi_{1}$ of interest. For instance, we can compare the ELG and LG distributions by testing $H_{0}:\alpha =1$ versus $H_{1}:\alpha \neq 1$. The ELG and Lindley distributions are compared by testing $H_{0}:\left(\alpha ,p\right)=\left(1,0\right)$ versus $H_{1}:\left(\alpha ,p\right)\neq \left(1,0\right)$.

### 3.2. Expectation-Maximization Algorithm

Dempster et al. [19] introduce an EM algorithm to estimate the parameters when some observations are treated as incomplete data. Suppose that $X=(X_{1},X_{2},\ldots ,X_{n})$ and $Z=(Z_{1},Z_{2},\ldots ,Z_{n})$ represent the observed and hypothetical data, respectively. Here, the hypothetical data can be thought of as missing data because $Z_{1},Z_{2},\ldots ,Z_{n}$ are not observable. We formulate the problem of finding the MLEs as an incomplete data problem, and thus, the EM algorithm is applicable to determine the MLEs of the ELG distribution. Let W=(X,Z) denote the complete data. To start this algorithm, define the pdf of each $\left(X_{i},Z_{i}\right)$ for i=1,…,n as

$$\begin{array}{cc} g(x,z,\alpha ,\theta ,p) & =\frac{\alpha \left(1-p\right)\theta^{2}z\left(1+x\right)}{\theta +1}e^{-\theta x}{\left(1-\frac{\theta +1+\theta x}{\theta +1}e^{-\theta x}\right)}^{\alpha -1}\\ & \;\;\;\;\times {\left[p-p{\left(1-\frac{\theta +1+\theta x}{\theta +1}e^{-\theta x}\right)}^{\alpha }\right]}^{z-1}. \end{array}$$

The E-step of an EM cycle requires the conditional expectation of $\left(Z|X,\alpha^{\left(r\right)},\theta^{\left(r\right)},p^{\left(r\right)}\right)$, where $\left(\alpha^{\left(r\right)},\theta^{\left(r\right)},p^{\left(r\right)}\right)$ is the current estimate of (α,θ,p) in the *r*the iteration. Please note that the pdf of *Z* given *X*, say g(z∣x), is given by

$$g\left(z|x\right)=\frac{z{\left[p-p{\left(1-\frac{\theta +1+\theta x}{\theta +1}e^{-\theta x}\right)}^{\alpha }\right]}^{z-1}}{{\left[1-p+p{\left(1-\frac{\theta +1+\theta x}{\theta +1}e^{-\theta x}\right)}^{\alpha }\right]}^{2}}.$$

Thus, the conditional expectation is given by

$$I\;E\left[Z|X,\alpha ,\theta ,p\right]=\frac{1+p\left[1-{\left(1-\frac{\theta +1+\theta x}{\theta +1}e^{-\theta x}\right)}^{\alpha }\right]}{1-p\left[1-{\left(1-\frac{\theta +1+\theta x}{\theta +1}e^{-\theta x}\right)}^{\alpha }\right]}.$$

The log-likelihood function $l_{c}\left(W;\alpha ,\theta ,p\right)$ of the complete data after ignoring the constants can be written as

(27) $$\begin{array}{cc} l_{c}\left(W;\alpha ,\theta ,p\right) & \propto \sum_{i=1}^{n}z_{i}+n\log\alpha +\sum_{i=1}^{n}\log(1+x_{i})+2n\log\theta -n\log\left(\theta +1\right)\\ & \;\;\;-\theta \sum_{i=1}^{n}x_{i}+n\log\left(1-p\right)+\left(\alpha -1\right)\sum_{i=1}^{n}\log\left(1-\frac{\theta +1+\theta x_{i}}{\theta +1}e^{-\theta x_{i}}\right)\\ & \;\;\;+\sum_{i=1}^{n}\left(z_{i}-1\right)\log\left[p-p{\left(1-\frac{\theta +1+\theta x_{i}}{\theta +1}e^{-\theta x_{i}}\right)}^{\alpha }\right]. \end{array}$$

Next the M-step involves the maximization of the pseudo log-likelihood function in (27). The components of the score function are given by

$$\begin{array}{cc} \frac{\partial l_{c}}{\partial \alpha } & =\frac{n}{\alpha }+\sum_{i=1}^{n}\log\left(1-\frac{\theta +1+\theta x_{i}}{\theta +1}e^{-\theta x_{i}}\right)-\sum_{i=1}^{n}\left(z_{i}-1\right)\frac{{\left(1-\frac{\theta +1+\theta x_{i}}{\theta +1}e^{-\theta x_{i}}\right)}^{\alpha }\log\left(1-\frac{\theta +1+\theta x_{i}}{\theta +1}e^{-\theta x_{i}}\right)}{1-{\left(1-\frac{\theta +1+\theta x_{i}}{\theta +1}e^{-\theta x_{i}}\right)}^{\alpha }},\\ \frac{\partial l_{c}}{\partial \theta } & =\frac{2n}{\theta }-\frac{n}{\theta +1}-\sum_{i=1}^{n}x_{i}+\left(\alpha -1\right)\sum_{i=1}^{n}\frac{\theta x_{i}e^{-\theta x}\left(1+x_{i}+\frac{1}{\theta +1}\right)}{\left(\theta +1\right)\left(1-\frac{\theta +1+\theta x_{i}}{\theta +1}e^{-\theta x_{i}}\right)}-\frac{\alpha \theta }{{\left(\theta +1\right)}^{2}}\\ & \;\;\;\times \sum_{i=1}^{n}\frac{\left(z_{i}-1\right)x_{i}\left(2+\theta +\theta x_{i}+x_{i}\right)e^{-\theta x_{i}}{\left(1-\frac{\theta +1+\theta x_{i}}{\theta +1}e^{-\theta x_{i}}\right)}^{\alpha -1}}{1-{\left(1-\frac{\theta +1+\theta x_{i}}{\theta +1}e^{-\theta x_{i}}\right)}^{\alpha }},\\ \frac{\partial l_{c}}{\partial p} & =-\frac{n}{1-p}+\sum_{i=1}^{n}\frac{z_{i}-1}{p}. \end{array}$$

For notational convenience, let

$$\tau_{i}^{\left(r\right)}\left(\theta \right)=1-\frac{\theta^{\left(r\right)}+1+\theta^{\left(r\right)}x_{i}}{\theta^{\left(r\right)}+1}e^{-\theta^{\left(r\right)}x_{i}},$$

for i=1,…,n. By replacing the missing *Z*’s with their conditional expectations $I\;E[Z|X,\alpha^{\left(r\right)},\theta^{\left(r\right)},p^{\left(r\right)}]$, we obtain an iterative procedure of the EM algorithm given by the following equations.

(28) $$\begin{array}{c} 0=\frac{n}{\alpha^{\left(r+1\right)}}+\sum_{i=1}^{n}\log\left(\tau_{i}^{\left(r+1\right)}\left(\theta \right)\right)-\sum_{i=1}^{n}\left(z_{i}-1\right)\frac{{\left(\tau_{i}^{\left(r+1\right)}\left(\theta \right)\right)}^{\alpha^{\left(r+1\right)}}\log\left(\tau_{i}^{\left(r+1\right)}\left(\theta \right)\right)}{1-{\left(\tau_{i}^{\left(r+1\right)}\left(\theta \right)\right)}^{\alpha^{\left(r+1\right)}}}, \end{array}$$

(29) $$\begin{array}{c} 0=\frac{2n}{\theta^{\left(r+1\right)}}-\frac{n}{\theta^{\left(r+1\right)}+1}-\sum_{i=1}^{n}x_{i}+\left(\alpha^{\left(r+1\right)}-1\right)\sum_{i=1}^{n}\frac{\theta^{\left(r+1\right)}x_{i}e^{-\theta^{\left(r+1\right)}x_{i}}\left(1+x_{i}+\frac{1}{\theta^{\left(r+1\right)}+1}\right)}{\left(\theta^{\left(r+1\right)}+1\right)\tau_{i}^{\left(r+1\right)}\left(\theta \right)}\\ \;\;\;-\frac{\alpha^{\left(r+1\right)}\theta^{\left(r+1\right)}}{{\left(\theta^{\left(r+1\right)}+1\right)}^{2}}\sum_{i=1}^{n}\frac{\left(z_{i}-1\right)x_{i}\left(2+\theta^{\left(r+1\right)}+\theta^{\left(r+1\right)}x_{i}+x_{i}\right)e^{-\theta^{\left(r+1\right)x_{i}}}{\left(\tau_{i}^{\left(r+1\right)}\left(\theta \right)\right)}^{\alpha^{\left(r+1\right)}-1}}{1-{\left(\tau_{i}^{\left(r+1\right)}\left(\theta \right)\right)}^{\alpha^{\left(r+1\right)}}},\\ p^{\left(r+1\right)}=1-\frac{n}{\sum_{i=1}^{n}z_{i}}, \end{array}$$

where

$$z_{i}=\frac{1+p^{\left(r\right)}\left[1-{\left(\tau_{i}^{\left(r\right)}\left(\theta \right)\right)}^{\alpha^{\left(r\right)}}\right]}{1-p^{\left(r\right)}\left[1-{\left(\tau_{i}^{\left(r\right)}\left(\theta \right)\right)}^{\alpha^{\left(r\right)}}\right]},$$

for i=1,…,n. Please note that some efficient numerical methods, such as the Newton-Raphson algorithm, are only needed for solving Equations (28) and (29).

### 3.3. Censored Maximum Likelihood Estimation

Censored data often occur in lifetime data analysis. Several popular mechanisms of censoring, such as type-I censoring and type-II censoring, have received much attention in the literature. The survival function of the ELG distribution has a simple closed-form expression, and therefore, it can be used in analyzing lifetime data in the presence of censoring. We briefly discuss the general case of multicensored data. Suppose that $n=n_{0}+n_{1}+n_{2}$ subjects of which

- $n_{0}$ is known to have failed at the times $t_{1},\ldots ,t_{n_{0}}$,
- $n_{1}$ is known to have failed into the interval $\left[s_{i-1},s_{i}\right]$ for $i=1,\ldots ,n_{1}$,
- $n_{2}$ is known to have survived at a time $r_{i}$ for $i=1,\ldots n_{2}$ but not observed any longer.

Please note that Type-I censoring and Type-II censoring are contained as particular cases of multicensoring above. The log-likelihood function of ϕ=(θ,α,p) of the ELG distribution for this multicensoring takes the form

$$\begin{array}{cc} l(\phi ;x) & =\sum_{i=1}^{n_{0}}\log(1+t_{i})+n_{0}\log\left[\frac{\alpha^{2}\theta^{2}\left(1-p\right)}{\theta +1}\right]-\theta \sum_{i=1}^{n_{0}}t_{i}+\left(\alpha -1\right)\log\left[1-\frac{\theta +1\theta t_{i}}{\theta +1}e^{-\theta t_{i}}\right]\\ & \;\;\;-\sum_{i=1}^{n_{0}}\log\left[1-p+p{\left(1-\frac{\theta +1\theta t_{i}}{\theta +1}e^{-\theta t_{i}}\right)}^{\alpha }\right]\\ & \;\;\;+\sum_{i=1}^{n_{1}}\log\left[\frac{{\left(1-\frac{\theta +1+\theta s_{i}}{\theta +1}e^{-\theta s_{i}}\right)}^{\alpha }}{1-p+p{\left(1-\frac{\theta +1+\theta s_{i}}{\theta +1}e^{-\theta s_{i}}\right)}^{\alpha }}-\frac{{\left(1-\frac{\theta +1+\theta s_{i-1}}{\theta +1}e^{-\theta s_{i-1}}\right)}^{\alpha }}{1-p+p{\left(1-\frac{\theta +1+\theta s_{i-1}}{\theta +1}e^{-\theta s_{i-1}}\right)}^{\alpha }}\right]\;dx\\ & \;\;\;+\sum_{i=1}^{n_{2}}\log\left[1-\frac{{\left(1-\frac{\theta +1+\theta r_{i}}{\theta +1}e^{-\theta r_{i}}\right)}^{\alpha }}{1-p+p{\left(1-\frac{\theta +1+\theta r_{i}}{\theta +1}e^{-\theta r_{i}}\right)}^{\alpha }}\right]. \end{array}$$

It is straightforward to derive the first derivatives of the log-likelihood function with respect to the three unknown parameters α, θ, and *p*. Thereafter, the MLEs of the unknown parameters can be obtained by setting the first derivatives equal to zero, i.e.,

$$\frac{\partial l(\phi ;x)}{\partial \theta }=\frac{\partial l(\phi ;x)}{\partial \alpha }=\frac{\partial l(\phi ;x)}{\partial p}=0.$$

Please note that the Newton-Raphson algorithm or other optimization algorithms may be employed to solve the above system of equations, because the MLEs of the unknown parameters cannot be obtained in closed-forms. Finally, the corresponding information matrix for ϕ is too complicated to be presented here.

## 4. Two Real-Data Applications

In this section, we illustrate the applicability of the ELG distribution using two real-data examples. We use the same data sets to compare the ELG distribution with the Gamma, Weibull, Lindley geometric (LG), Weibull geometric (WG) distributions, whose densities are given by

(i)  Gamma(β,α)
  $$\begin{array}{cc} f_{1}\left(x\right) & =\frac{1}{\Gamma (\beta )}\alpha^{\beta }x^{\beta -1}e^{-\alpha x},\;\;\beta >0,\;\;\alpha >0; \end{array}$$
(ii)  Weibull(β,λ)
  $$\begin{array}{cc} f_{2}\left(x\right) & =\frac{\alpha }{\beta }{\left(\frac{x}{\beta }\right)}^{\alpha -1}e^{-{\left(x/\beta \right)}^{\alpha }},\;\;\beta >0,\;\;\alpha >0; \end{array}$$
(iii)  LG(θ,p)
  $$\begin{array}{cc} f_{3}\left(x\right) & =\frac{\theta^{2}}{\theta +1}\left(1-p\right)\left(1+x\right)e^{-\theta x}{\left[1-\frac{p(\theta +1+\theta x)}{\theta +1}e^{-\theta x}\right]}^{-2},\;\;\theta >0,\;\;p<1, \end{array}$$
(iv)  WG(α,β,p)
  $$\begin{array}{cc} f_{4}\left(x\right) & =\alpha \beta^{\alpha }\left(1-p\right)x^{\alpha -1}e^{-{\left(\beta x\right)}^{\alpha }}{\left[1-pe^{-{\left(\beta x\right)}^{\alpha }}\right]}^{-2},\;\;\alpha >0,\;\;\beta >0,\;\;p<1, \end{array}$$

for x>0, respectively. To compare the ELG distribution with the four distributions listed above, we advocate the Akaike information criterion (AIC), the Bayesian information criterion (BIC), and the AIC with a correction (AICc) for the two-real data sets. In addition, we apply two formal goodness-of-fit tests: the Cramér-von Mises ($W^{*}$) and Anderson-Darling ($A^{*}$) statistics to further verify which distribution fits better to the data; see, for example, [5,20], among others. The smaller the value of the considered criterion, the better the fit to the data.

**Figure 4:**
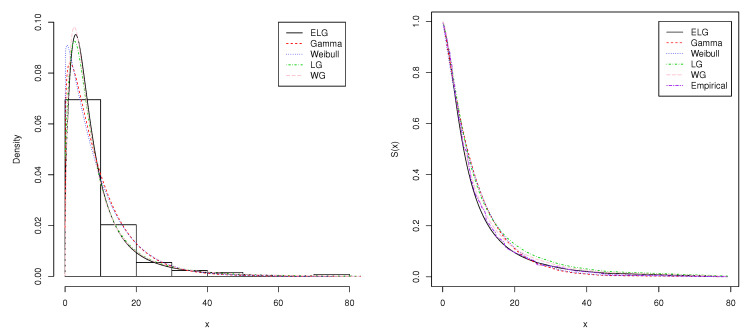
Plots of the estimated density and survival function of the fitted models for the first data set.

The first data set is about the remission time (in months) of a random sample of 128 bladder cancer patients. This data set presented in Table 1 was studied by [21] in fitting the extended Lomax distribution and [22] for the modified Weibull geometric distribution. Table 2 shows the MLEs of the parameters, AIC, BIC, and AICc for the ELG, Gamma, Weibull, LG, and WG distributions for the first data set. We observe from Table 2 that the ELG distribution and its special case LG provide an improved fit over other distributions that are commonly used for fitting lifetime data. The plots of the fitted probability density and survival function are also shown in Figure 4. Please note that the density and survival functions of the ELG distribution seem to be better than Gamma, Weibull, and WG density and survival functions. In addition, we observe from the values of goodness-of-fit tests in Table 3 that the ELG distribution fits the current data better than other distributions under consideration.

As mentioned in Section 3.1, we can adopt the LR statistic to compare between the ELG distribution and its special submodels. For example, the LR statistic for testing between the LG and ELG distributions (i.e., $H_{0}:\alpha =1$ versus $H_{1}:\alpha \neq 1$) is ω=0.5645 and the corresponding *p*-value is 0.4525. Thus, we fail to reject $H_{0}$ and conclude that there is no statistical difference between the fits to this data using the ELG and its submodel LG. This is quite reasonable because the estimate of α in the ELG model is $\hat{\alpha }=1.0792$, which is close to 1 in the LG model.

**Figure 5:**
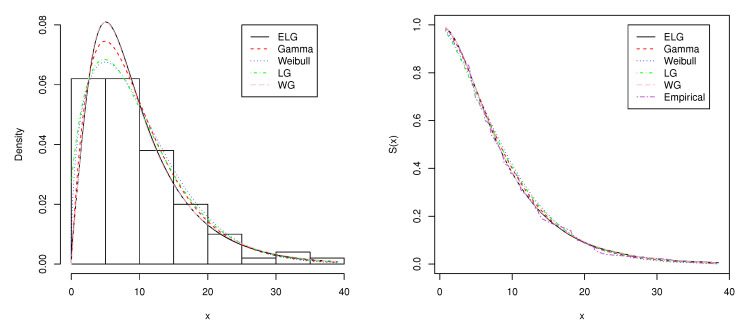
Plots of the estimated density and survival function of the fitted models for the second data set.

In the second data set, we consider the waiting time (in minutes) before service of 100 bank customers. The data are presented in Table 4. This data set was used by [12] in fitting the Lindley distribution. Table 5 shows the MLEs of the parameters, AIC, BIC, and AICc for the ELG, Gamma, Weibull, LG, and WG distributions for the second data set. Table 5 indicates that the ELG distribution is still a strong competitor to other lifetime distributions. In addition, the plots of the fitted probability density and survival function are shown in Figure 5. Please note that the ELG and WG distributions perform identically and that the empirical and fitted five survival curves almost overlap for this data set, supporting that the ELG distribution fits this data at least as good as the four alternative distributions. In addition, we observe from the values of goodness-of-fit tests in Table 6 that the ELG distribution fits the current data better than the Gamma, Weibull, and LG distributions and is comparable with the WG distribution.

## 5. Concluding Remarks

In this paper, we introduced the exponentiated Lindley geometric distribution, which generalizes the LG distribution due to [14] and the Lindley distribution proposed by [23]. We have studied various statistical properties of the new distribution. Estimations of the unknown parameters of the distribution are discussed based on the maximum likelihood estimation and an EM algorithm is provided for estimating the parameters. In an ongoing project, we study and Bayesian inference of these parameters and results will be reported elsewhere.

## Figures and Tables

**Table 1:** The first data set: the remission time (in months) of a random sample of 128 bladder cancer patients.

0.08	2.09	3.48	4.87	6.94	8.66	13.11	23.63	0.20	2.23
3.52	4.98	6.97	9.02	13.29	0.40	2.26	3.57	5.06	7.09
9.22	13.80	25.74	0.50	2.46	3.64	5.09	7.26	9.47	14.24
25.82	0.51	2.54	3.70	5.17	7.28	9.74	14.76	26.31	0.81
2.62	3.82	5.32	7.32	10.06	14.77	32.15	2.64	3.88	5.32
7.39	10.34	14.83	34.26	0.90	2.69	4.18	5.34	7.59	10.66
15.96	36.66	1.05	2.69	4.23	5.41	7.62	10.75	16.62	43.01
1.19	2.75	4.26	5.41	7.63	17.12	46.12	1.26	2.83	4.33
5.49	7.66	11.25	17.14	79.05	1.35	2.87	5.62	7.87	11.64
17.36	1.40	3.02	4.34	5.71	7.93	11.79	18.10	1.46	4.40
5.85	8.26	11.98	19.13	1.76	3.25	4.50	6.25	8.37	12.02
2.02	3.31	4.51	6.54	8.53	12.03	20.28	2.02	3.36	6.76
12.07	21.73	2.07	3.36	6.93	8.65	12.63	22.69		

**Table 2:** MLEs of the fitted models, AIC, BIC, and AICc for the first data set.

Model		Parameters		AIC	BIC	AICc
Gamma	$\hat{\alpha }$ = 0.1252	$\hat{\beta }=1.1726$		830.7356	836.4396	830.8316
Weibull	$\hat{\alpha }$ = 1.0478	$\hat{\beta }$ = 9.5607		832.1738	837.8778	832.2698
LG	$\hat{\theta }$ = 0.0742	$\hat{p}=0.8898$		823.1859	833.742	823.2819
WG	$\hat{\alpha }$ = 1.6042	$\hat{\beta }$ = 0.0286	$\hat{p}$ = 0.9362	826.1842	834.7403	826.3777
ELG	$\hat{\alpha }$ = 1.0792	$\hat{\theta }$ = 0.0699	$\hat{p}$ = 0.9204	824.6214	833.1775	824.8149

**Table 3:** Goodness-of-fit tests for the first data set.

	Statistic	
Model	$W^{*}$	$A^{*}$
Gamma	0.11988	0.71928
Weibull	0.13136	0.78643
LG	0.05374	0.33827
WG	0.01493	0.09939
ELG	0.01389	0.09498

**Table 4:** The second data set: the waiting time (in minutes) before service of 100 bank customers.

0.8	0.8	1.3	1.5	1.8	1.9	1.9	2.1	2.6	2.7
2.9	3.1	3.2	3.3	3.5	3.6	4.0	4.1	4.2	4.2
4.3	4.3	4.4	4.4	4.6	4.7	4.7	4.8	4.9	4.9
5.0	5.3	5.5	5.7	5.7	6.1	6.2	6.2	6.2	6.3
6.7	6.9	7.1	7.1	7.1	7.1	7.4	7.6	7.7	8.0
8.2	8.6	8.6	8.6	8.8	8.8	8.9	8.9	9.5	9.6
9.7	9.8	10.7	10.9	11.0	11.0	11.1	11.2	11.2	11.5
11.9	12.4	12.5	12.9	13.0	13.1	13.3	13.6	13.7	13.9
14.1	15.4	15.4	17.3	17.3	18.1	18.2	18.4	18.9	19.0
19.9	20.6	21.3	21.4	21.9	23.0	27.0	31.6	33.1	38.5

**Table 5:** MLEs of the fitted models, AIC, BIC, and AICc for the second data set.

Model		Parameters		AIC	BIC	AICc
Gamma	$\hat{\alpha }$ = 0.2033	$\hat{\beta }=2.0089$		638.6002	643.8106	638.724
Weibull	$\hat{\alpha }$ = 1.4585	$\hat{\beta }$ = 10.9553		641.4614	646.6717	641.5851
LG	$\hat{\theta }$ = 0.2027	$\hat{p}=-0.2427$		641.8269	647.0372	641.9506
WG	$\hat{\alpha }$ = 1.9789	$\hat{\beta }$ = 0.0501	$\hat{p}$ = 0.82132	639.9084	647.7239	640.1584
ELG	$\hat{\alpha }$ = 1.4602	$\hat{\theta }$ = 0.1725	$\hat{p}$ = 0.5385	640.3108	648.1263	640.5608

**Table 6:** Goodness-of-fit tests for the second data set.

	Statistic	
Model	$W^{*}$	$A^{*}$
Gamma	0.02761	0.18225
Weibull	0.06294	0.39624
LG	0.05374	0.33827
WG	0.01706	0.12365
ELG	0.01801	0.12665
